# Transcriptome Analysis of Choroid and Retina From Tree Shrew With Choroidal Neovascularization Reveals Key Signaling Moieties

**DOI:** 10.3389/fgene.2021.654955

**Published:** 2021-05-10

**Authors:** Jie Jia, Dandan Qiu, Caixia Lu, Wenguang Wang, Na Li, Yuanyuan Han, Pinfen Tong, Xiaomei Sun, Min Wu, Jiejie Dai

**Affiliations:** ^1^Institute of Medical Biology, Chinese Academy of Medical Science and Peking Union Medical College, Kunming, China; ^2^Scientific Research Laboratory Center, The First Affiliated Hospital of Kunming Medical University, Kunming, China; ^3^Kunming Medical University, Kunming, China; ^4^Yunnan Eye Institute, The Second People’s Hospital of Yunnan, Kunming, China

**Keywords:** choroidal neovascularization, transcriptome sequencing, bioinformatics, tree shrew, signal transduction

## Abstract

Pathological neovascularization in choroid, a leading cause of blindness, is a characteristic of many fundus diseases, such as diabetic retinopathy and age-related macular degeneration. The present study aimed to elucidate the key signaling pathways in choroidal neovascularization (CNV) by analyzing the mRNA profiles of choroid and retina in tree shrews with CNV. We induced choroidal angiogenesis by laser photocoagulation in 15 tree shrews and obtained mRNA profiles of their choroids and retinas by high-throughput transcriptome sequencing. Hierarchical cluster analysis, weighted gene co-expression network analysis (WGCNA), protein-protein interaction (PPI) network analysis, hematoxylin and eosin (HE) staining, CD31 immunohistochemistry (IHC), and reverse transcription quantitative PCR (RT-qPCR) were performed. After laser photocoagulation, we obtained a total of 350 differentially expressed genes (DEGs) in the choroid, including 59 genes in Module-FASN (“ME-FASN”) module and 28 genes in Module-RPL (“ME-RPL”) module. A total of 69 DEGs in retina, including 20 genes in Module-SLC (“ME-SLC”) module. Bioinformatics analysis demonstrated that DEGs in choroid were mainly involved in membrane transport; DEGs in “ME-RPL” were prominent in pathways associated with IgA production, antigen presentation, and cell adhesion molecules (CAMs) signaling. DEGs in “ME-FASN” were involved in fatty acid metabolism and PPAR signaling pathway, while DEGs in “ME-SLC” were involved in GABAergic synapse, neuroactive life receptor interaction, cholinergic synapse, and retrograde endocannabinoid signaling pathway. PPI network analysis demonstrated that the ribosomal protein family genes (*RPL31, RPL7, RPL26L1,* and *RPL19*) are key factors of “ME-RPL,” acyl-CoA superfamily genes (*ACACA, ACAT1, ACAA2,* and *ACACB*) and *FASN* are key factors of “ME-FASN” and superfamily of solid carrier genes (*SLC17A6, SLC32A1, SLC12A5,* and *SLC6A1*) and complement genes (*C4A, C3,* and *C2*) are key factors of “ME-SLC.” In conclusion, the present study discovered the important signal transductions (fatty acid metabolic pathway and CAMs signaling) and genes (ribosomal protein family and the complement system) in tree shrew CNV. We consider that our findings hold implications in unraveling molecular mechanisms that underlie occurrence and development of CNV.

## Introduction

Choroidal neovascularization (CNV) is the formation of new blood vessels in the choroid. The blood vessels form/develop between the retinal pigment epithelium (RPE) and Bruch’s membrane. They extend through the retinal neuroepithelial layer eventually forming a fibrous vascular tissue. CNV often occurs in the macular area, thereby causing macular hemorrhage and serous exudation under the retina. CNV is a common characteristic of many fundus diseases, such as age-related macular degeneration, high myopic maculopathy, central exudative chorioretinopathy, and diabetic retinopathy; it renders grave vision impairment in the affected individuals.

Although the pathogenesis of CNV remains poorly understood, it is believed to involve a variety of cell growth factors, such as the vascular endothelial growth factor (VEGF), basic fibroblast growth factor (bFGF), and platelet-derived growth factor (PDGF), available in the local microenvironment (Ming et al., 2011). Moreover, inflammatory cells and cytokines are also involved in its pathogenesis, while tumor necrosis factor-α (TNF-α), interleukins 6 (IL6), and intercellular cell adhesion molecules 1 (ICAM-1) released by macrophages and neutrophils promote the occurrence and development of CNV (Jin et al., 2010).

The early stage of CNV is characterized by changes in the retinal microenvironment along with production of VEGF by the RPE and photoreceptors (PRs; Nagineni et al., 2012). Further, VEGF promotes the migration of macrophages to the Bruch’s membrane, eventually leading to proteolytic degradation of the membrane (Grossniklaus et al., 2002). However, the key molecular mechanisms underlying CNV and its signaling between retina and choroid remain unclear.

Animal models are important tools in investigating pathogenesis of CNV. The most commonly used method to establish a CNV model is laser-induced selective destruction of photoreceptors, RPE cells, Bruch’s membrane, and choroidal capillaries. Destruction of the Bruch’s membrane stimulates a series of damage repair processes that leads to the formation of new blood vessels (Archer and Gardiner, 1981; ElDirini et al., 1991; Yang et al., 2006; Hoerster et al., 2012; Liu et al., 2013; Kunbei et al., 2014; Poor et al., 2014). Pathogenesis of CNV in humans is same as that in animals, wherein an injury to the Bruch’s membrane-RPE-choroidal complex leads to an imbalance between angiogenic and inhibitory factors, resulting in a series of neovascularization processes, such as endothelial cell proliferation and migration, and lumen formation.

Animals that are most often used for CNV experiments are monkeys, rabbits, and mice. Although the monkey model manifests changes/characteristics similar to that by humans, their application in CNV studies is limited owing to their high cost of maintenance and low incidence of CNV (Archer and Gardiner, 1981; Kunbei et al., 2014). Further, the CNV rabbit model fails to exhibit typical leakage characteristics (less than 30%) as observed by FFA. In addition, retinal vascular circulation of mice and rabbits is disparate from that of humans; therefore, mice and rabbits are not ideal models for CNV studies (Zhu et al., 1989; ElDirini et al., 1991; Tamai et al., 2002; Yang et al., 2006; Hoerster et al., 2012; Liu et al., 2013; Poor et al., 2014). Tree shrew is a small mammal sharing evolutionary and anatomical similarities with primates. Tree shrews have a well-developed visual system, with cone cells accounting for 96% of all the photosensitive cells. These mammals have good color vision as well as stereoscopic vision. In recent years, tree shrews have been used for investigating visual development and ophthalmic diseases (Lin et al., 2013; Jia and Dai, 2019). The retina of tree shrews is mainly composed of cone cells, as mentioned above, and exhibits functions similar to that of human retina. The unique retinal structure of tree shrew provides a good basis for investigating the disease process of human CNV.

In the present study, we aimed to investigate the signal transduction in choroid and retina in a tree shrew CNV model. We found that the genes of ribosomal protein family, superfamily of acyl-CoA, and solute carrier family were differentially expressed. Our findings imply that fatty acid metabolic pathway and CAM pathway play key roles in tree shrew CNV.

## Materials and Methods

### Experimental Animals

Fifteen adult tree shrews (*Tupaia belangeri chinensis*) with healthy eyes (seven females and eight males, aged 2–3 years, weighing 110–130 g) were collected from the Tree Shrew Germplasm Resource Center, Institute of Medical Biology, Chinese Academy of Medical Sciences, Kunming, China. The experimental animal production licenses were SCXK (Dian) K2018-0002 and use license SYXK (Dian) K2018-0002. All animal experiments were approved by the Animal Ethics Committee of the Institute of Medical Biology, Chinese Academy of Medical Science (approval number: DWSP201803019). The tree shrews were randomly divided into three groups (7, 21, and 30 days after laser photocoagulation) such that each group comprised five tree shrews (10 eyes per group, all left eyes were used as the experimental group and all right eyes as the control group).

### Laser-Induced CNV Model

The animals were anesthetized by intraperitoneal injection of 0.3% pentobarbital sodium (0.6 mg/kg), and the body temperature was maintained by a heating pad. Five minutes before laser photocoagulation, compound tropicamide eye drops were used to disperse the pupil of both eyes. Eyes were anesthetized with two drops of 4 mg/ml oxybuprocaine hydrochloride eye drops (Santen Pharmaceutical Co., Ltd., China) and carbomer eye drops (Dr. Gerhard Mann, Chem.-Pharm. Fabrik GmbH, Germany) was used to prevent corneal dryness. After the animals were fixed, laser photocoagulation (Lumenis, United States) was performed at 1 PD around the optic disc, 15 spots in total. The laser wavelength was 647.1 nm, diameter of the spots was 50 μm, and the exposure time was 0.01–0.05 s. Energy of the laser was adjusted according to the retinal reaction. The effective spots were marked by the formation of bubbles without bleeding after photocoagulation. Eyes with ruptured capillaries small blood vessels were not used for subsequent experiments (Fundus camera: TOPCON, United States).

### Hematoxylin and Eosin Staining

Eyeballs of tree shrew corneas were fixed with FAS (Servicebio, China) for 24 h, and 3-um-thick of were cut. The eyeball sections were dehydrated, cleared, embedded, and made into sections. The sections were used for hematoxylin and eosin (HE) staining and CD31 immunohistochemistry (IHC). The pathological changes were described by a section scanner (Mantra, United States).

### CD31 Immunohistochemistry

In order to confirm the occurrence of CNV after photocoagulation, IHC for CD31 was conducted.

Antigen repair was performed in citric acid sodium buffer solution (ph6.0; Servicebio, China). Sections were incubated in 3% hydrogen peroxide at room temperature for 25 min in dark to block endogenous peroxidase, and then blocked with 3% BSA.

Sections were incubated in 1:300 dilution of Anti-CD31 Rabbit pAb (Servicebio, China) at 4°C overnight, and then incubated in 1:200 dilution of HRP conjugated Goat Anti-Rabbit IgG (H + L; Servicebio, China) for 50 min at room temperature. Color development was performed using DAB (Servicebio, China). The result of CD31 IHC was observed under microscope (Mantra, United States), and inform was used for processing the image. Microvessel density (MVD) were counted refer to Weidner et al. (1993).

### RNA Sequencing of Retina and Choroid of Tree Shrew CNV Model

#### Total RNA Extraction

Tree Shrews were euthanized by injection of 2% pentobarbital sodium (2 mg/kg). Tree shrews were disinfected and the eyeballs were rinsed with iodophor. The canthus and the conjunctiva were cut. The conjunctiva and fascia were separated so that the sclera was fully exposed. The optic nerve was cut and then the eyeball was removed.

The eyeballs were suspended in PBS in clean bench. After the cornea was cut, the lens and vitreum were separated. The retina-choroid-sclera was transferred to RNase-free water (TaKaRa, Japan) to isolate the retina (soft and transparent). Since the choroid was fragile and easy to fall off, the choroid-sclera was transferred to TRIzol and choroid (brown) was isolated.

Total RNA was extracted from the collected samples using TRIzol according to the standard protocol (Invitrogen, United States). Purity of the extracted RNA was spectrophotometrically quantified with NanoPhotometer (Implen, United States). RNA integrity was determined using Bioanalyzer 2100 system (Agilent Technologies, United States). In case the RNA content was insufficient (RNA < 2 μg), the sample was mixed with other samples of the same group.

#### cDNA Library Preparation and Sequencing

First-strand DNA was synthesized using random primers and M-MuLV reverse transcriptase (RNase H-). Second-strand DNA was synthesized by DNA polymerase I and RNase H, wherein dTTPs were replaced by dUTPs. Further, the dU-containing second-strand cDNA was degraded by the USER enzyme, and the cDNA was PCR-amplified to obtain a library.

The cDNA was quantified using Qubit 2.0, and insert size of the library was determined by diluting the library to 1 ng/μl and subjecting to Agilent 2100 system. Effective concentration of the cDNA library was quantified accurately by qPCR (effective library concentration > 2 nM) to ensure quality of the cDNA library. Twenty-six DNA libraries were constructed. The libraries were sequenced on an Illumina HiSeq 4000 platform at the Novogene Bioinformatics Institute (Beijing, China).

### Differential Expression of mRNA in the Retina and Choroid

#### Cluster Analysis

Subread package (Liao et al., 2014) was used to filter out the sequences with adapter, poly-n > 10%, and low quality of RNA sequencing to get clean data. TopHat v2.0.9 (Kim et al., 2013) was used to align clean data with tree shrew genome sequences (http://www.treeshrewdb.org/, Accession number: CRP000902), genome annotation in http://www.treeshrewdb.org/data/Chinese_treeshrew_function_annotation_information_2.0.txt.gz. The mapped fragments were spliced using the Cufflinks V2.1.1 (Trapnell et al., 2010). Gene expression level was quantitatively analyzed by Fragments Per Kilobase of exon model per Million (FPKM; mapped fragments; Bray et al., 2016). After statistical analyses (Anders and Huber, 2010; Robinson et al., 2010; Love et al., 2014), differentially expressed genes (DEGs) in the retina and choroid were selected at | log_2_ (fold change) | > 1 and Padj < 0.05.

We perform heatmap clustering with distance methods of pre-defined distance method (1-pearson), and clustering method of clustering function [cluster_rows = diana(mat) and cluster_columns = agnes *t*(mat)] from cluster package (Gu et al., 2016).

#### GO and KEGG Analyses

In order to determine the functions of the DEGs, clusterProfiler was used to analyze gene ontoloagy (GO) function and kyoto encyclopedia of genes and genomes (KEGG) pathway enrichment. GO analyzes the biological processes, cellular components, and molecular functions. KEGG analysis determines the signal pathways enriched by the DEGs, and it is expected to find the biological pathways that play key roles in tree shrew CNV. Threshold of GO/KEGG enrichment was considered significant at Padj < 0.05.

#### Protein-Protein Interaction Network Construction

STRING database^1^ provides comprehensive information about interactions between proteins. In the present study, STRING was used to generate PPI networks among the differentially expressed mRNAs based on interactions with combined scores > 0.4. Additionally, Cytoscape was used to visualize the network, and PPI scores > 800 were considered for functional enrichment analysis of the modules.

#### Weighted Gene Co-expression Network Analysis

Hierarchical cluster tree was established using WGCNA based on the correlation of gene expressions at the Pearson correlation coefficient 0.8 and soft threshold (power) 9. Functions were considered relevant when the genes demonstrated a high degree of co-expression correlation in a module. The modules were clustered and the correlation heatmap among modules was constructed to evaluate the connectivity between two genes within the module according to the module eigenvalues.

We analyzed the expression trends of DEGs in the modules during different courses of CNV.

#### Reverse Transcription Quantitative PCR

The results of RNA sequencing were validated by reverse transcription quantitative PCR (RT-qPCR) using CFX96 PCR system (Bio-Rad, United States) and One Step TB Green PrimeScript PLUS RT-PCR Kit (TaKaRa, Japan). Five significantly differentially expressed transcripts were selected (*FASN, ACACB, ACAT1, ACAA2*, and *IL18*; Table 1). The reaction mixture (25 μl) contained 12.5 μl 2 × One Step TB Green RT-PCR Buffer, 0.5 μl PrimeScript PLUS RTase Mix, 1.5 μl TaKaRa Ex Taq HS Mix, 6 μl RNase-free ddH_2_O, 2 μl RT-qPCR primers, 0.5 μl ROX reference dye, and 1 μg total RNA.

**Table 1:** Reverse transcription quantitative PCR (RT-qPCR) primer sequences and amplification conditions.

Transcript	Primers	Sequence (5' to 3')	Annealing temperature (°C)	Product length (bp)
*ACACB*	Forward	CATCCGCGCCATTATCAG	60	171
	Reverse	GGCACGAAGTTGAGGAAG		
*ACAT1*	Forward	CACTGCCAGCCACTAAACTTG	60	119
	Reverse	TCCTTCGCCTCCTTGTAGAAC		
*ACAA2*	Forward	CACTGCTACTGACTTGAC	60	103
	Reverse	CTGCATGACATTGCCTAC		
*IL18*	Forward	TTAGAGGTCTGGCTGTAG	61	111
	Reverse	CGCTGATATTGTCAGGAG		
*FASN*	Forward	CTGCTGCTGAAACCTACC	60	200
	Reverse	CAGCCGTCTGTGTTAGTG		
*GAPDH*	Forward	CTTCAACTCTGGCAAGGT	60 – 61	279
	Reverse	AAGATGGTGATGGACTTCC		

The reaction was performed at the following conditions: initial cDNA synthesis at 45°C for 5 min; initial denaturation at 95°C for 10 s; 40 cycles of denaturation at 95°C for 5 s, annealing and extension at 60–61°C for 30 s; 95°C for 15 s, 60°C for 60 s, and 95°C for 15 s for the dissolution curve. The internal reference gene was *GAPDH*. Each experiment was performed in triplicate. PCR primers and amplification conditions used in this study are shown in Table 1.

### Statistical Analysis

Statistical analyses were performed using SPSS 20.0. All data are expressed as mean ± SEM. *p* < 0.05 was considered statistically significant. Student’s *t*-test was used for analyzing RT-qPCR and MVD results. GraphPad Prism 8.0 was used to prepare the graphs.

## Results

### Pathology of the Tree Shrews CNV

**Figure 1:**
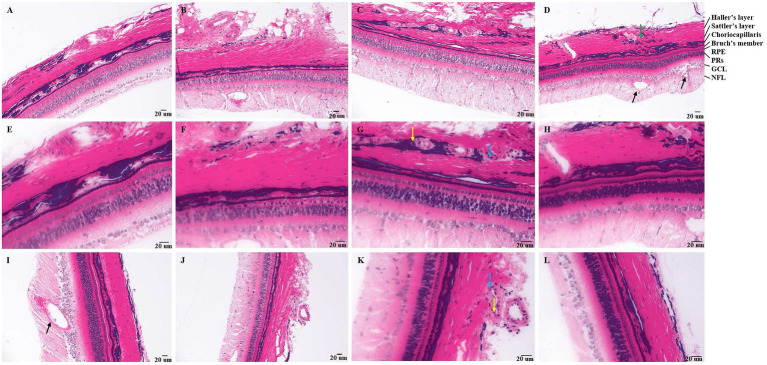
Pathology of tree shrews with choroidal neovascularization (CNV). [**A** and **B** (200×), **E** and **F** (400×)] Pathology of tree shrew CNV after 7 days of laser photocoagulation. [**C** and **D** (200×), **G** and **H** (400×)] Pathology of tree shrew after CNV 14 days of laser photocoagulation. [**J** (200×), **I** and **K** (400×)] Pathology of tree shrew CNV after 21 days of laser photocoagulation. [**L** (400×)] Intact retina and choroid with clear structure of normal tree shrew. The yellow arrow indicates neutrophils, the blue arrow indicates macrophages, the black arrow indicates neovascularization, and the green arrow indicates loose haller’s and sattler’s layer in choroid. RPE, Retinal pigment epithelium; PRs, Photoreceptor; GCL, ganglion cell; and NFL, nerve fiber layer.

In the control groups, tree shrews have intact retina (Figure 1L). After 7 days of photocoagulation, retinal edema (Figures 1B,F), damaged haller’s layer in choroid (Figures 1A,E) was observed. After 14 days of photocoagulation, neovascularization in retina leads to destruction of ganglion cell (GCL) layer and disorder of nerve fiber layer (NFL) layer. CNV was developed, and neovascularization surrounded by neutrophils, macrophages, and fibroblasts was observed found in choroid (Figures 1C,G). Haller’s layer and sattler’s layer was loose in choroid (Figures 1D,H). After 30 days of photocoagulation, retinal neovascularization was developed (Figure 1I). Neutrophils and macrophages were seen around the neovascularization in choroid. Damaged choroidal structure was not recover (Figures 1J,K).

### IHC for CD31

**Figure 2:**
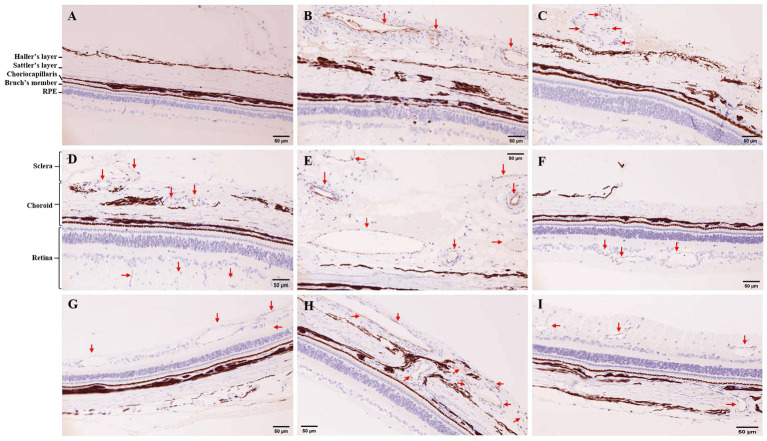
Immunohistochemistry (IHC) for CD31 in tree shrew retina and choroid (200×). **(A)** IHC in control group. **(B,C)** IHC in 7 days of photocoagulation group. **(D,E,F)** IHC in 14 days of photocoagulation group. **(G,H,I)** IHC in 21 days of photocoagulation group. The red arrow indicates neovascularization. RPE, Retinal pigment epithelium.

The normal tree shrews have complete retina, choroid and sclera with distinct layers, and no neovascularization was observed (Figure 2A). After 7 days of photocoagulation, retinal edema was observed in the photocoagulation sites, and the choroid structure was destroyed. Neovascularization was observed in the subchoroid (Figure 2C) and scleral (Figure 2B), but no neovascularization was observed in the choroid and retina. After 14 days of photocoagulation, retinal edema was still observed in the photocoagulation sites. Neovascularization with small size was obvious in the retina (Figures 2D,F) and choroid (Figure 2D), while the neovascularization in the sclera was larger than that in 7 days of photocoagulation group (Figure 2E). After 21 days of photocoagulation, increased retinal (Figure 2I) and choroid (Figure 2H) neovascularization in the photocoagulation sites. The lumen of retinal neovascularization was larger than that in 7 days of photocoagulation group (Figure 2G).

Microvessel density in laser photocoagulation group was higher than that in control group (*p* < 0.05). MVD in 14 days of photocoagulation group (7.00 ± 1.00) were higher than that in 7 days of photocoagulation group (3.33 ± 0.58). However, MVD were no significant difference between 14 days of photocoagulation group and 21 days of photocoagulation group (5.33 ± 2.31).

### RNA Sequencing

Raw reads were generated from 36 samples, including 18 choroids and 18 retinas. The average clean data of each sample were not less than 7 GB. The average number of clean reads was 46,719,819; Q20 was higher than 97%; Q30 was higher than 94%.

### Quantitative Assessment by PCR

**Figure 3:**
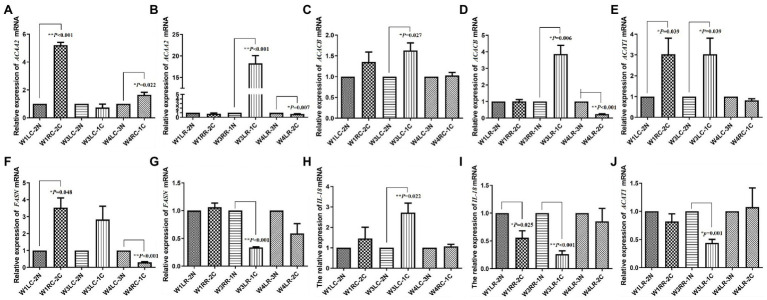
Validation of transcript expression by quantitative PCR (qPCR). **(A,B)** The expression of *ACAA2* mRNA in tree shrew choroid and retina, respectively; **(C,D)** The expression of *ACACB* mRNA in tree shrew choroid and retina, respectively; **(E,J)** The expression of *ACAT1* mRNA in tree shrew choroid and retina, respectively; **(F,G)** The expression of *FASN* mRNA in tree shrew choroid and retina, respectively; **(H,I)** The expression of *IL18* mRNA in tree shrew choroid and retina, respectively. Data are presented as mean ± SEM (*n* = 3). *GAPDH* was used as a housekeeping internal control. Transcript expression was quantified relative to the expression level of *GAPDH* by 2^-ΔΔCT^ method. 0.001 ≤ ^*^p < 0.05; ^**^p < 0.001.

Relative expressions of *FASN, ACACB, ACAT1, ACAA2*, and *IL18* are shown in Figure 3. The expression of *ACAA2* was significantly upregulated after 7 days (*p* < 0.001) and downregulated after 30 days (*p* = 0.022) of photocoagulation in choroid (Figure 3A), whereas it was upregulated after 21 days (*p* < 0.001) and downregulated after 30 days (*p* = 0.007) of photocoagulation in the retina (Figure 3B).

The expression of *ACACB* was significantly upregulated after 21 days (*p* = 0.027) of photocoagulation in choroid (Figure 3C), and upregulated after 21 days (*p* = 0.006), but downregulated after 30 days (*p* < 0.001) of photocoagulation in retina (Figure 3D).

The expression of *FASN* was upregulated after 7 days (*p* = 0.048) in the choroid (Figure 3F), but downregulated after 21 days (*p* < 0.001) and 30 days (*p* < 0.001) of photocoagulation in the retina (Figure 3G).

The expression of *IL18* was upregulated after 21 days (*p* < 0.022) and 30 days (*p* = 0.022) of photocoagulation in choroid (Figure 3H), but downregulated after 7 days (*p* < 0.025) and 21 days (*p* < 0.001) of photocoagulation in the retina (Figure 3I).

The expression of *ACAT1* was upregulated after 7 days (*p* = 0.039) and 21 days (*p* = 0.039) of photocoagulation in the choroid (Figure 3E), but downregulated after 21 days (*p* = 0.001) of photocoagulation in the retina (Figure 3J). The qPCR results were consistent with the RNA sequencing data.

### Cluster Analysis of DEGs

**Figure 4:**
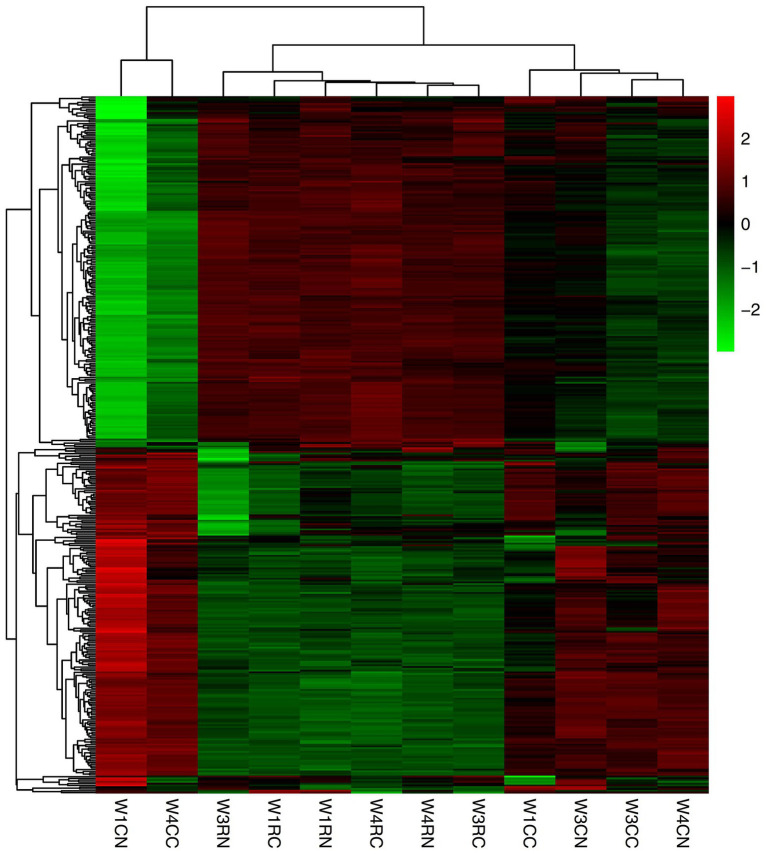
Cluster analysis of differentially expressed mRNAs. Red indicates upregulated mRNAs; green indicates downregulated mRNAs. W1CC is choroid after 7 days of laser photocoagulation, W1CN is another choroid collected from the same tree shrew as W1CC. W3CC and W4CC are choroids after 21 and 30 days of laser photocoagulation, respectively. W3CN and W4CN are choroids collected from the same tree shrews as W3CC and W4CC, respectively. W1RC is retina after 7 days of laser photocoagulation, W1RN is another retina collected from the same tree shrew as W1RC. W3RC and W4RC are retina after 21 and 30 days of laser photocoagulation, respectively. W3RN and W4RN are retina collected from the same tree shrews as W3RC and W4RC, respectively.

The mRNA expression levels were estimated with FPKM. In the choroid, 335 mRNAs, 9 mRNAs, and 6 mRNAs were differentially expressed after 7, 21, and 30 days of laser photocoagulation, respectively. In the retina, 6, 56, and 7 mRNAs were differentially expressed after 7, 21, and 30 days of photocoagulation, respectively. A cluster analysis of the differentially expressed mRNAs was conducted and the results are shown as heatmaps in Figure 4.

### WGCNA

#### Hierarchical Cluster Tree

**Figure 5:**
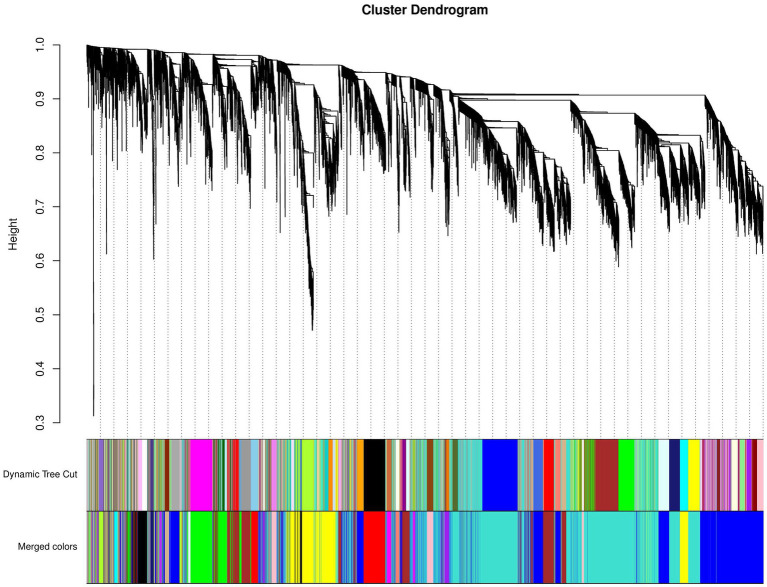
Cluster dendrogram of differentially expressed mRNAs. Upper portion of the figure is the gene cluster tree. A leaf represents a gene while the branches represent different gene modules. Different colors in the middle portion of the dynamic tree cut represent different modules. Merged colors (lowermost portion) represent the merged modules calculated when the dissimilarity coefficient < 0.25. Yellow tree represents Module-FASN (“ME-FASN”) module; Blue tree represents Module-RPL (“ME-RPL”) module; Turquoise tree represents Module-SLC (“ME-SLC”) module.

We obtained a total of 18 gene modules and found that the DEGs were mainly clustered in the Module-RPL (“ME-RPL”), Module-SLC (“ME-SLC”), and Module-FASN (“ME-FASN”) modules. These modules contained 37, 184, and 91 significant DEGs, respectively (Figure 5).

#### Heatmap of Inter-Module Correlation

**Figure 6:**
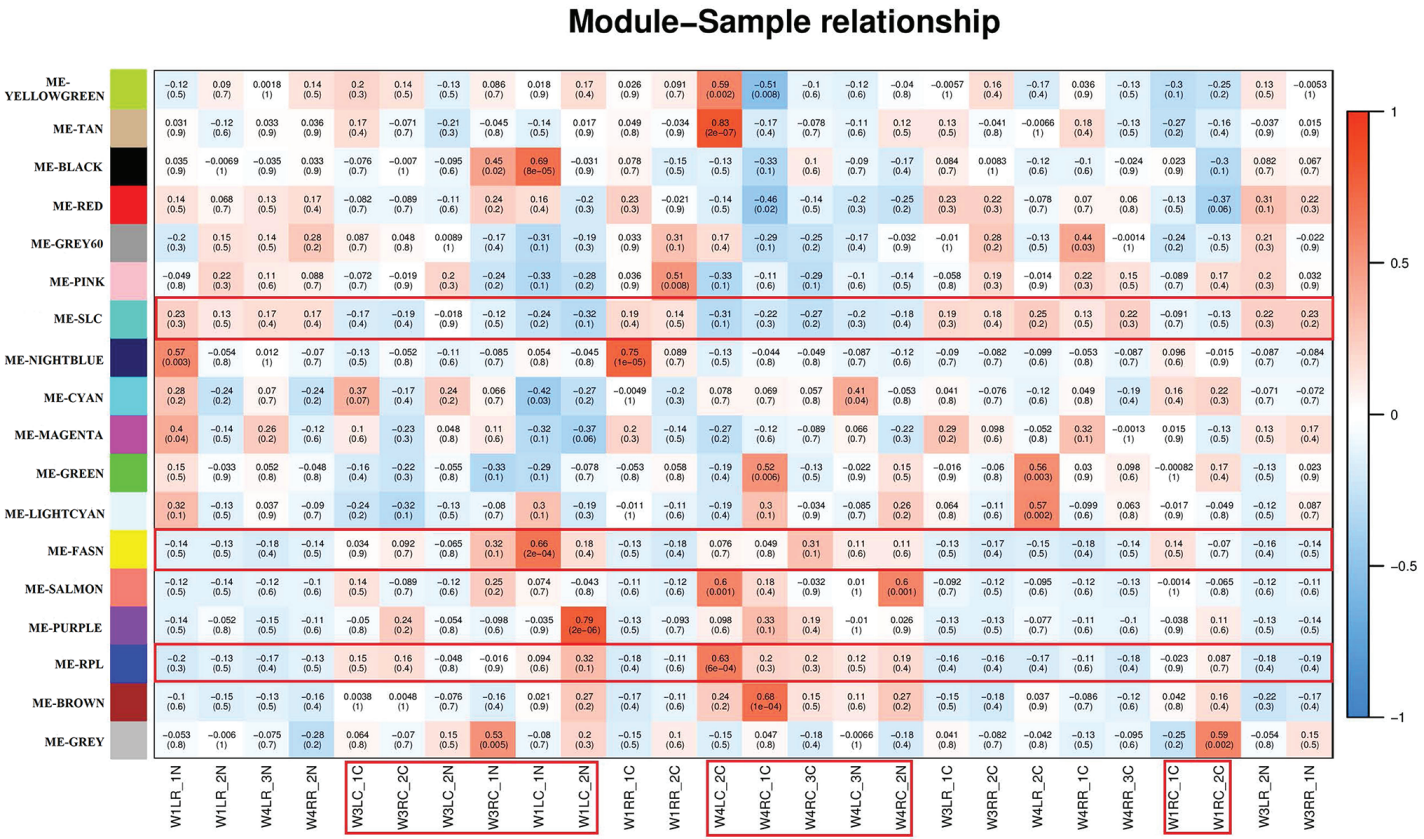
Module – Sample relationships. The abscissa is the sample and the ordinate is the module. The numbers represent correlation between the module and the sample. Closer the value is to 1, stronger the positive correlation between the module and the sample; closer to −1, stronger the negative correlation. The number in brackets represents value of *p*, smaller the value, and higher the significance. The red specimen is choroid of the tree shrews.

Correlation analysis between the modules and samples showed that “ME-RPL,” “ME-FASN,” and choroid are highly (and positively) correlated, while “ME-SLC” and retina are highly (and positively) correlated, as depicted in Figure 6.

#### Cluster Heatmap of the Gene Modules

**Figure 7:**
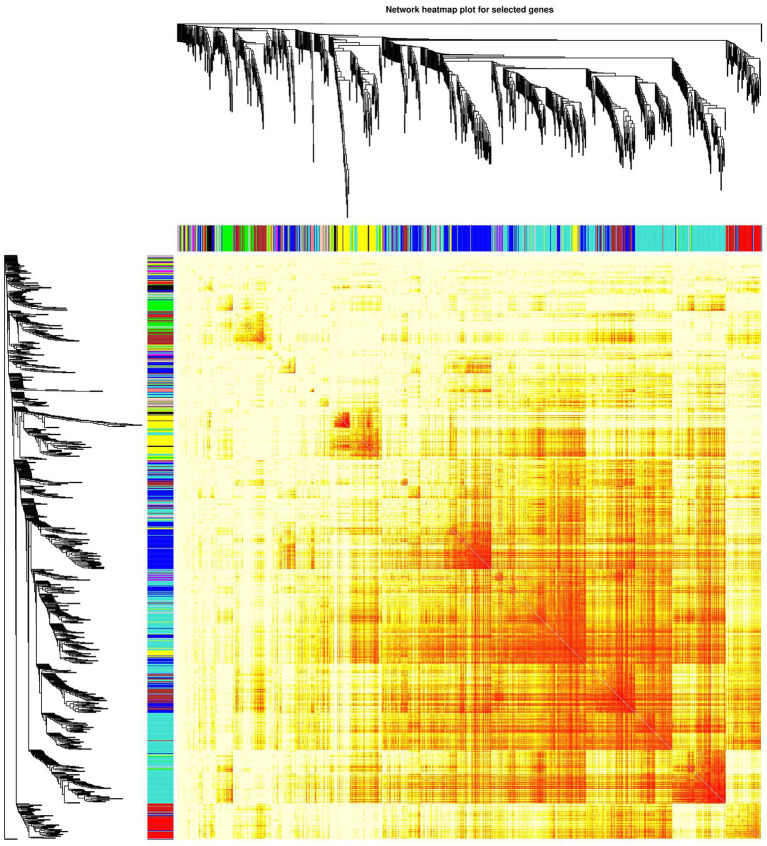
Network heatmap of the gene modules. The tree represents a module (top and left), and the branch represents a gene. Darker the color of the dot (white → yellow → red), stronger the connectivity between the two genes corresponding to the row and column. Yellow tree represents “ME-FASN” module; Blue tree represents “ME-RPL” module; Turquoise tree represents “ME-SLC” module.

Analysis of interactions among the gene modules revealed that when the color of the region within and between the three modules is evident, the genes in these modules have a high degree of association. It is suggested that “ME-RPL,” “ME-SLC,” and “ME-FASN” modules interact closely with each other. The results in the form of heatmap visualization are shown in Figure 7.

#### Expression Patterns of the Gene Modules

**Figure 8:**
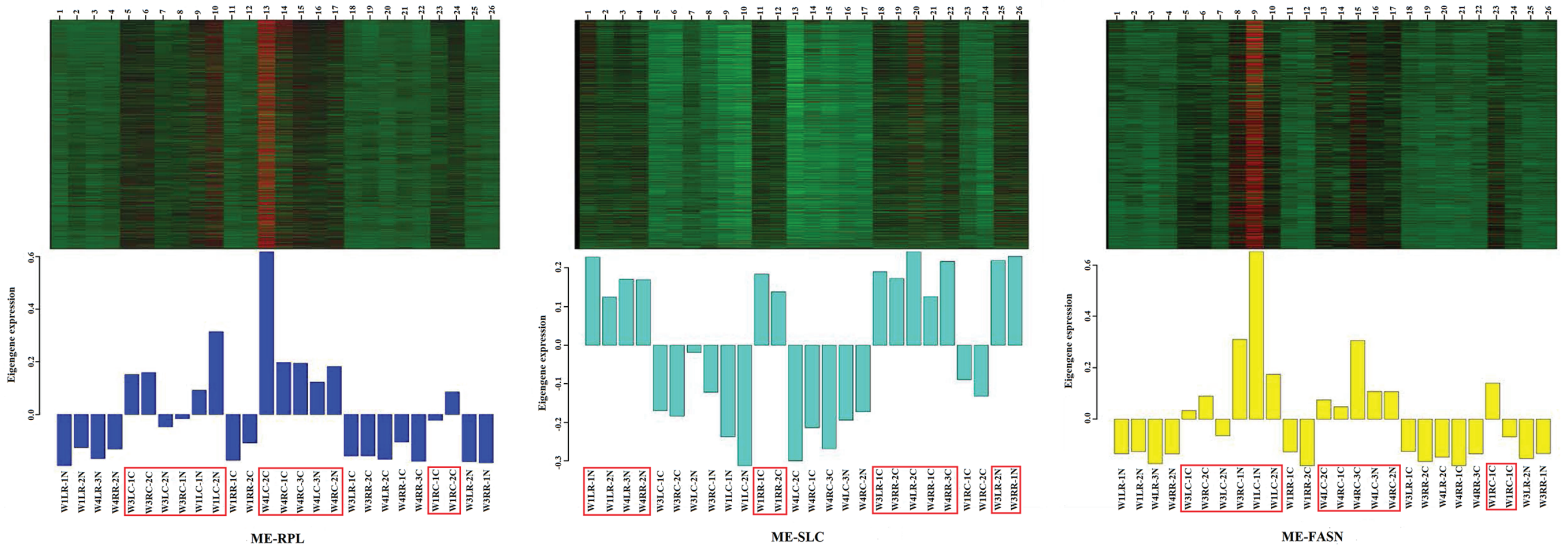
Heatmap visualization of eigenvalues of “ME-RPL,” “ME-SLC,” and “ME-FASN” modules. Upper portion of the figure shows the gene expression heatmaps. Red indicates upregulated; green indicates downregulated. Lower portion of the figure shows the module eigenvalues in different samples, the displayed gene is upregulated/downregulated in the respective module.

In order to analyze the expression trend of the genes in different modules, the module gene expression pattern was constructed. The results showed that “ME-RPL” genes were downregulated in the retina but upregulated in choroid; “ME-SLC” genes were upregulated in the retina but downregulated in choroid; and “ME-FASN” genes were downregulated in retina but upregulated in choroid, as shown in Figure 8.

#### GO and KEGG Analyses of DEGs

**Figure 9:**
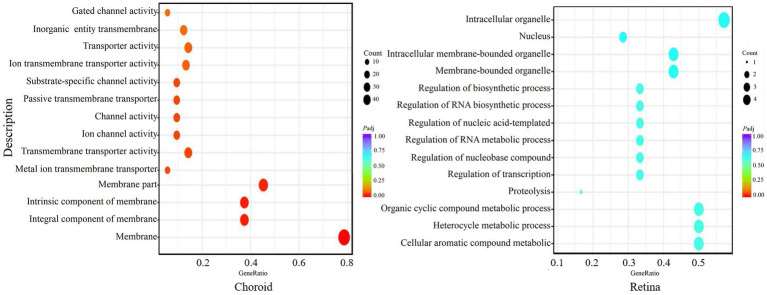
GO enrichment of the significant differentially expressed genes (DEGs) in the choroid and retina. The abscissa shows the rich factors and the ordinate represents the pathways. Size of each point indicates the number of candidate target genes in the GO term, and the color of each point corresponds to the value of *p* after correction.

GO analysis showed that the differentially expressed mRNAs in the choroid were mainly involved in membrane transport processes, while there was no significant enrichment for differentially expressed mRNA in the retina (Figure 9).

**Figure 10:**
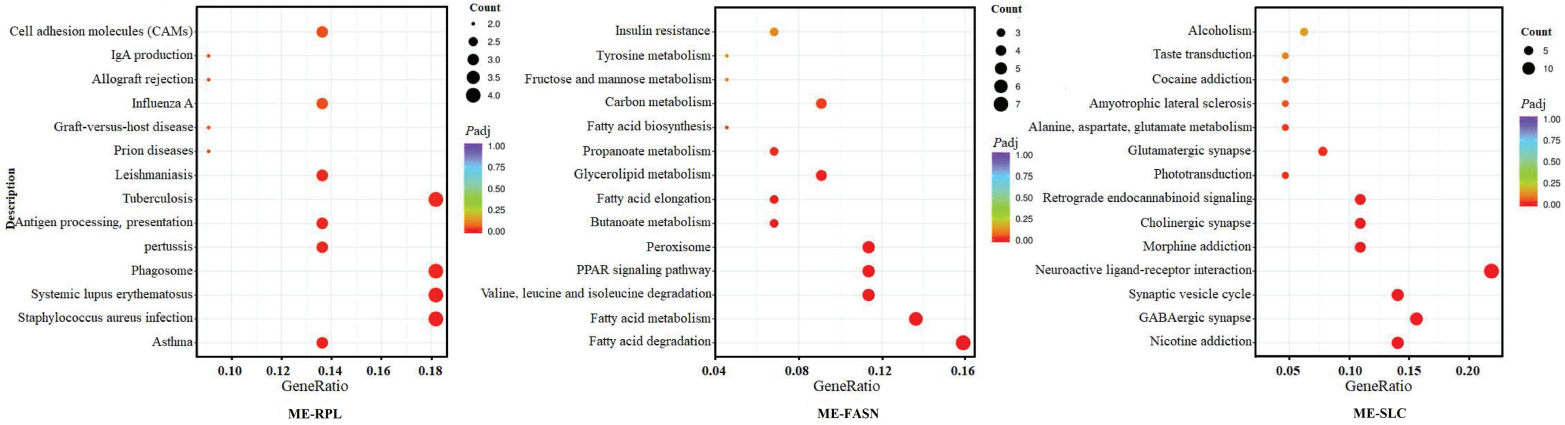
KEGG enrichment of the significant differentially expressed mRNAs in “ME-RPL,” “ME-FASN,” and “ME-SLC.” The abscissa shows the rich factors and the ordinate represents the pathways. Size of each point indicates the number of candidate target genes in the pathway, and the color of each point corresponds to the value of *p* after correction.

KEGG analysis showed that “ME-RPL” genes were enriched in IgA production, antigen presentation, and CAMs pathways. “ME-FASN” genes are enriched in fatty acid metabolism and PPAR signaling pathway, while “ME-SLC” genes were enriched in GABAergic synapse formation/development, neuroactive ligand-receptor interaction, cholinergic synapse, and retrograde endocannabinoid signaling pathway (Figure 10).

#### PPI Network Analysis

**Figure 11:**
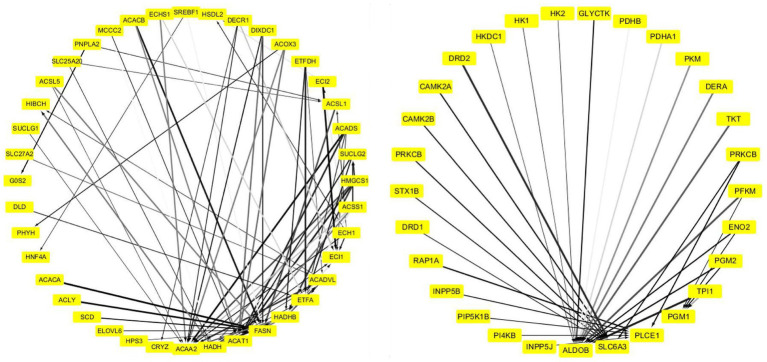
PPI network of the DEGs in “ME-FASN.” Grayscale value of each line correlates with the strength of the interaction between the proteins.

**Figure 12:**
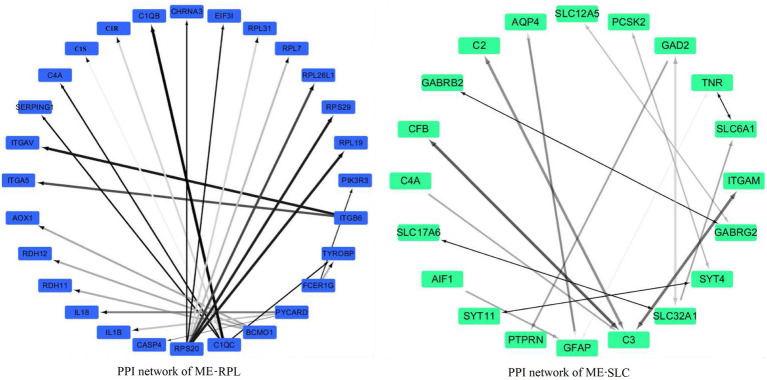
PPI networks of the DEGs in “ME-RPL” and “ME-SLC.” Grayscale value of each line correlates with the strength of the interaction between the proteins.

The PPI network of “ME-FASN” consisted of 69 genes and 102 interactions, including superfamily of acyl-CoA (*ACACA, ACAT1, ACAA2, ACACB, ACADS,* and *ACADVL*) and superfamily of solid carrier genes (*SLC6A3, SLC25A20,* and *SLC27A2*; Figure 11). “ME-RPL” consisted of 28 genes and 23 interactions, including ribosomal protein family (*RPL31, RPL7, RPL26L1,* and *RPL19*), complement genes (*C4A, C1S,* and *C1R*), and integrin genes (*ITGA5, ITGB6,* and *ITGAV*; Figure 11). “ME-SLC” consisted of 20 genes and 17 interactions, including superfamily of solid carrier genes (*SLC17A6, SLC32A1, SLC12A5,* and *SLC6A1*) and complement genes (*C4A, C3,* and *C2*; Figure 12).

## Discussion

Pathogenesis of CNV is influenced by the choroid microenvironment that is composed of extracellular matrix, cytokines, PDGF, fibroblast growth factors, tumor necrosis factor-α (TNF-α), pigment epithelium-derived growth factor, interleukins, the complement system, ephrins, and angiopoietins (Lambert et al., 2016). Reportedly, CNV-related signaling pathways mainly include the VEGF pathway, transforming growth factor (TGF)-β/Smad pathway (Wang et al., 2017), Wnt pathway (Zhou et al., 2010), sonic hedgehog (Shh) pathway (Yang et al., 2012; Holliday et al., 2013), and notch pathway (Dou et al., 2016). VEGF is the most important cytokine for neovascularization that can stimulate the proliferation and migration of vascular endothelial cells and promote capillary lumen formation (Jie et al., 2004). TNF-α contributes to CNV by upregulating VEGF production in RPE cells through ROS-dependent activation of β-catenin signaling (Haibo et al., 2016). Wnt3a has been shown to activate β-catenin, upregulate VEGF and TNF-α, and increase oxidative stress (Zhou et al., 2010) while WNT7A/B promotes CNV (Joseph et al., 2018). FGF21 inhibits TNF-α expression but does not alter VEGFA expression in neovascular eyes, and FGF21 administration suppresses pathological retinal neovascularization and CNV in mice (Fu et al., 2017). Following Bruch’s membrane injury, RPE cells release IL6, IL8, and growth factors (TGF-β) thereby exerting strong chemotactic effects on macrophages and neutrophils involved in the neovascularization (Hollyfield et al., 2008; Zarranz-Ventura et al., 2013). It is reported that the lack of TGF-β signaling in retinal microglia can cause retinal degeneration and aggravate CNV (Ma et al., 2019). Several studies have found that TGF-β significantly enhances VEGF secretion, vascular permeability, and extracellular matrix remodeling on its own or in concert with other cytokines, such as TNF-α (Walshe et al., 2009; Suzuki et al., 2012).

In the present study, we showed that the expression of *IL18* increased significantly after 21 days of laser photocoagulation in the choroid, although it decreased significantly after 7 and 21 days of laser photocoagulation in the retina. In mouse model of CNV, the expression of *IL18* decreased significantly in RPE-choroid tissue (Choudhary et al., 2015). It has been reported that *IL18* regulates pathological retinal neovascularization. In addition, IL18 inhibits the formation of experimental CNV through the stimulation of interferon-γ and thrombospondin (Xiao and Wu, 2016). IL18 reduce CNV development in the nonhuman primate eye, and inhibits vascular leakage in a mouse model of spontaneous neovascularization (Doyle et al., 2015). However, the mechanism of IL18 inhibiting CNV formation remains unclear and needs to be further investigated.

In present study, genes in “ME-RPL” had a high correlation with choroid. Moreover, KEGG analysis showed that “ME-RPL” genes were significantly enriched in CAMs pathway. CAMs are associated with neovascularization (Dong et al., 2018), wherein ICAM-1 is closely related to the occurrence and development of CNV (Gao and He, 2015). However, the role of CAMs in CNV remains to be fully understood.

There were significant differences in the expression of ribosomal protein family genes in the “ME-RPL” module, and the interaction among proteins in “ME-RPL” was close. It has been reported that RPL17 inhibits the growth of vascular smooth muscle cells and silencing *RPL17* promotes proliferation of these cells (Smolock et al., 2012). It is suggested that the ribosomal protein family may be involved in the regulation of vascular growth. Concurrent with previous studies, we found that the ribosomal protein family may be involved in the regulation of CNV. However, the underlying mechanism requires further in-depth research.

In the “ME-FASN” module, fatty acid synthase (*FASN*) was the center of PPI network. KEGG analysis showed that many “ME-FASN” DEGs were enriched in the fatty acid metabolic, GABAergic synapse, neuroactive life receptor interaction, cholinergic synapse, and retrograde endocannabinoid pathways. *FASN* regulates tumor angiogenesis by altering secretion and activity of VEGF (Seguin et al., 2012). This study suggests that the acylation involved in fatty acid metabolism may be related to CNV. The acyl-CoA superfamily genes (*ACACA, ACACB, ACAA2*, and *ACADVL*) in “ME-FASN” module were significantly differentially expressed and had strong interactions with *FASN*. In mouse model of CNV, the *ACACB* and *ACADVL* gene was significantly differentially expressed (Choudhary et al., 2015), which correlate with present study. The present study indicates that fatty acid metabolic pathway and acyl-CoA superfamily genes may play an important role in the occurrence and development of CNV. It is reported that FASN knockdown in endothelial cells elevated malonyl-CoA levels, and reduced pathological ocular neovascularization (Bruning et al., 2018). The expression levels of acyl-CoA oxidase 1 (Acox1), fatty acid binding protein 4 (Fabp4) is associated with the progression of retinal pathological neovascularization in a murine model (Tomita et al., 2019). *SLC27A2* and *C3* gene in “ME-SLC” were significantly differentially expressed in CNV tree shrew models and CNV mouse model (Choudhary et al., 2015). The result above demonstrated that the transcriptome profile in CNV tree shrew model and in CNV mouse model is consistency. In addition, the result of huvec cell (human umbilical vein endothelial cell) high throughput sequencing in NCBI GEO database (Popovic et al., 2020) showed that *IL18*, *FASN*, *ACADS*, *SLC25A20*, *RPL7*, and *RPL26L1* gene was significantly differentially expressed after treated with DAND5, it indicated that these gene may related to the developmental and pathological ocular angiogenesis. Furthmore, there are 12 genes (*ACACB*, *IL18*, *RPS20*, *ACSL1*, *ACADVL*, *HMGCS1*, *ECI1*, *TKT*, *EIF3I*, *PYCARD*, *CASP4*, and *CFB*) were differentially expressed in tree shrew CNV, mouse CNV (Choudhary et al., 2015), and huvec cell neovascularization models (Popovic et al., 2020).

In conclusion, we constructed the interaction network of “ME-RPL” involved in CAMs pathway and “ME-FASN” genes involved in fatty acid metabolism in choroid, “ME-SLC” genes involved in GABAergic synapse in retina during CNV in tree shrews. Our findings hold implications in unraveling molecular mechanisms that underlie occurrence and development of CNV. Although with cone cells accounting for 96% of all the photosensitive cells, tree shrew not have discernible macula. The problem of lack established transgenic technology for tree shrews need to be solved, and the role of CAMs and fatty acid metabolic pathway needs further investigations to elucidate CNV pathogenesis at the molecular level.

## Data Availability Statement

The datasets presented in this study can be found in online repositories. The name of the repository and accession number can be found below: The European Molecular Biology Laboratory’s European Bioinformatics Institute (EMBL-EBI) ArrayExpress, https://www.ebi.ac.uk/arrayexpress/, E-MTAB-10198.

## Ethics Statement

The animal study was reviewed and approved by Animal Ethics Committee of the Institute of Medical Biology, Chinese Academy of Medical Science (Ethics approval number: DWSP201803019).

## Author Contributions

JJ, DQ, and JD conceived and designed this study. JJ, DQ, and NL performed the experiments. XS, WW, CL, YH, and PT collected the samples. JJ and DQ interpreted the data and drafted and edited the manuscript. The study was performed under the supervision of JD and MW. All authors contributed to the article and approved the submitted version.

### Conflict of Interest

The authors declare that the research was conducted in the absence of any commercial or financial relationships that could be construed as a potential conflict of interest.

## Footnotes

^1^https://string-db.org/

## Supplementary Material

Supplementary Figure 1 Symptoms of tree shrews with choroidal neovascularization (CNV) after laser photocoagulation. **(B)** Representative fundus angiograph of normal tree shrew. **(B)** Representative fundus angiograph of tree shrew with laser photocoagulation. **(C,D)**: FFA/ICGA of normal tree shrew. **(E,F)**: FFA/ICGA of tree shrew after 7 days of laser photocoagulation. **(G,H)**: FFA/ICGA of tree shrew after 30 days of laser photocoagulation.

## References

[ref1] Anders S. Huber W. (2010). Differential expression analysis for sequence count data. Genome Biol. 11:R106. 10.1186/gb-2010-11-10-r106, PMID: 20979621 PMC3218662

[ref2] Archer D. B. Gardiner T. A. (1981). Morphologic flurescein angiographic, and light microscopic features of experimental choroidal neovascularization. Am J. Ophthalmol. 91, 297–311. 10.1016/0002-9394(81)90281-6 6163359

[ref3] Bray N. L. Pimentel H. Melsted P. Pachter L. (2016). Near-optimal probabilistic RNA-seq quantification. Nat. Biotechnol. 34, 525–527. 10.1038/nbt.3519, PMID: 27043002

[ref4] Bruning U. Morales-Rodriguez F. Kalucka J. Goveia J. Taverna F. Queiroz K. C. S.. (2018). Impairment of angiogenesis by fatty acid synthase inhibition involves mTOR Malonylation. Cell Metab. 28, 866–880.e15. 10.1016/j.cmet.2018.07.019, PMID: 30146486 PMC8057116

[ref500] Choudhary M. Kazmin D. Hu P. Thomas R. S. McDonnell D. P. Malek G. (2015). Aryl hydrocarbon receptor knock-out exacerbates choroidal neovascularization via multiple pathogenic pathways. J. Pathol. 235, 101–102. 10.1002/path.4433, PMID: 25186463 PMC4277859

[ref5] Dong Z. J. Shi Y. N. Zhao H. J. Li N. Ye L. Zhang S.. (2018). Sulphonated formononetin induces angiogenesis through vascular endothelial growth factor/cAMP response element-binding protein/early growth response 3/vascular cell adhesion molecule 1 and Wnt/β-catenin signaling pathway. Pharmacology 101, 76–85. 10.1159/000480662 29131133

[ref6] Dou G. R. Li N. Chang T. F. Zhang P. Gao X. Yan X. C.. (2016). Myeloid-specific blockade of notch signaling attenuates choroidal neovascularization through compromised macrophage infiltration and polarization in mice. Sci. Rep. 6:28617. 10.1038/srep28617 27339903 PMC4919651

[ref7] Doyle S. L. López F. J. Celkova L. Brennan K. Mulfaul K. Ozaki E.. (2015). IL-18 immunotherapy for neovascular AMD: tolerability and efficacy in nonhuman primates. Invest. Ophthalmol. Vis. Sci. 56, 5424–5430. 10.1167/iovs.15-17264, PMID: 26284546

[ref8] ElDirini A. A. Ogden T. E. Ryan S. J. (1991). Subretinal endophotocoagulation. A new model of subretinal neovascularization in the rabbit. Retina 11, 244–249. 10.1097/00006982-199111020-00010, PMID: 1925091

[ref9] Fu Z. J. Gong Y. Liegl R. Wang Z. X. Liu C. H. Meng S. S.. (2017). FGF21 administration suppresses retinal and choroidal neovascularization in mice. Cell Rep. 18, 1606–1613. 10.1016/j.celrep.2017.01.014, PMID: 28199833 PMC5328201

[ref10] Gao X. Y. He S. Z. (2015). Dynamic expression of intercellular adhesion molecule-1 in laser-induced choroidal neovascularization in brown Norway rats. Chin. J. Exp. Ophthalmol. 33, 1103–1107. 10.3760/ema.j.issn.2095-0160.2015.12.011

[ref11] Grossniklaus H. E. Ling J. X. Wallace T. M. Dithmar S. Lawson D. H. Cohen C.. (2002). Macrophage and retinal pigment epithelium expression of angiogenic cytokines in choroidal neovascularization. Mol. Vis. 8, 119–126. PMID: 11979237

[ref12] Gu Z. Eils R. Schlesner M. (2016). Complex heatmaps reveal patterns and correlations in multidimensional genomic data. Bioinformatics 32, 2847–2849. 10.1093/bioinformatics/btw313, PMID: 27207943

[ref13] Haibo W. Xiaokun H. Erika S. Hartnett M. E. (2016). TNF-α mediates choroidal neovascularization by upregulating VEGF expression in RPE through ROS-dependent β-catenin activation. Mol. Vis. 22, 116–128. PMID: 26900328 PMC4736754

[ref14] Hoerster R. Muether P. S. Vierkotten S. Schröder S. Kirchhof B. Fauser S. (2012). In-vivo and ex-vivo characterization of laser-induced choroidal neovascularization variability in mice. Graefes Arch. Clin. Exp. Ophthalmol. 250, 1579–1586. 10.1007/s00417-012-1990-z, PMID: 22419036

[ref15] Holliday E. G. Smith A. V. Cornes B. K. Buitendijk G. H. S. Jensen R. A. Sim X. L.. (2013). Insights into the genetic architecture of early stage age-related macular degeneration: a genomewide association study meta-analysis. PLoS One 8:e53830. 10.1371/journal.pone.0053830, PMID: 23326517 PMC3543264

[ref16] Hollyfield J. G. Bonilha V. L. Rayborn M. E. Yang X. P. Shadrach K. G. Lu L.. (2008). Oxidative damage-induced inflammation initiates age-related macular degeneration. Nat. Med. 14, 194–198. 10.1038/nm1709, PMID: 18223656 PMC2748836

[ref17] Jia J. Dai J. J. (2019). Advantages and challenges of tree shrews in biomedical research. Lab. Anim. Comp. Med. 39, 3–8. 10.26914/c.cnkihy.2019.068189

[ref18] Jie Z. Yusheng W. Yannian H. (2004). Formation of choroidal neovascularization and its inhibition. Rec. Adv. Ophthalmol. 24, 57–60. 10.13389/j.cnki.rao.2004.01.028

[ref19] Jin L. Yuhua H. Xin Z. (2010). Relation between inflammation and choroidal neovascularization. Rec. Adv. Ophthalmol. 30, 293–296. 10.13389/j.cnki.rao.2010.03.008

[ref20] Joseph B. L. Abdoulaye S. Luke A. W. Santeford A. Nudleman E. Nakamura R.. (2018). WNT7A/B promote choroidal neovascularization. Exp. Eye Res. 174, 107–112. 10.1016/j.exer.2018.05.033, PMID: 29864439 PMC6110966

[ref21] Kim D. Pertea G. Trapnell C. Pimentel H. Kelley R. Salzberg S. L. (2013). TopHat2: accurate alignment of transcriptomes in the presence of insertions, deletions and gene fusions. Genome Biol. 14:R36. 10.1186/gb-2013-14-4-r36, PMID: 23618408 PMC4053844

[ref22] Kunbei L. Chenjin J. Shu T. Yunfan X. Rui H. Jian G. (2014). The study of laser-induced choroidal neovascularization in rhesus monkeys. Chin. J. Ophthalmol. 50, 203–208. 10.3760/cma.j.issn.0412-4081.2014.03.010 24841817

[ref23] Lambert N. G. ElShelmani H. Singh M. K. Mansergh F. C. Wride M. A. Padilla M.. (2016). Risk factors and biomarkers of age-related macular degeneration. Prog. Retin. Eye Res. 54, 64–102. 10.1016/j.preteyeres.2016.04.003, PMID: 27156982 PMC4992630

[ref25] Liao Y. Smyth G. K. Shi W. (2014). featureCounts: an efficient general purpose program for assigning sequence reads to genomic features. Bioinformatics 30, 923–930. 10.1093/bioinformatics/btt656, PMID: 24227677

[ref26] Lin X. Yun Z. Bin L. Lü L. B. Chen C. S. Chen Y. B.. (2013). Tree shrews under the spot light: emerging model of human diseases. Zool. Res. 34, 59–69. 10.3724/SP.J.1141.2013.02059 23572354

[ref27] Liu T. Hui L. Wang Y. S. Guo J. Q. Li R. Su J. B.. (2013). In-vivo investigation of laser-induced choroidal neovascularization in rat using spectral-domain optical coherence tomography (SD-OCT). Graefes Arch. Clin. Exp. Ophtalmol. 251, 1293–1301. 10.1007/s00417-012-2185-3 23114625

[ref28] Love M. I. Huber W. Anders S. (2014). Moderated estimation of fold change and dispersion for RNA-seq data with DESeq2. Genome Biol. 15, 1–21. 10.1186/s13059-014-0550-8 PMC4302049 25516281

[ref29] Ma W. X. Silverman S. M. Lian Z. Villasmil R. Campos M. Amaral J.. (2019). Absence of TGFb signaling in retinal microglia induces retinal degeneration and exacerbates choroidal neovascularization. Elife Sci. 8:e42049. 10.7554/eLife.42049, PMID: 30666961 PMC6342522

[ref30] Ming A. Fang Y. Dai L. (2011). Recent advance on pathogenesis of choroidal neovascularization. J. Clin. Ophthalmol. 19, 351–354. 10.3969/j.issn.1006-8422.2011.04.038

[ref31] Nagineni C. N. Kommineni V. K. William A. Detrick B. Hooks J. J. (2012). Regulation of VEGF expression in human retinal cells by cytokines: implications for the role of inflammation in age-related macular degeneration. J. Cell. Physiol. 227, 116–126. 10.1002/jcp.22708, PMID: 21374591 PMC12039479

[ref32] Poor S. H. Qiu Y. Fassbender E. S. Shen S. Woolfenden A. Delpero A.. (2014). Reliability of the mouse model of choroidal neovascularization induced by laser photocoagulation. Invest. Ophthalmol. Vis. Sci. 55, 6525–6534. 10.1167/iovs.14-15067, PMID: 25205860

[ref24] Popovic N. Hooker E. Barabino A. Flamier A. Provost F. Buscarlet M.. (2020). COCO/DAND5 inhibits developmental and pathological ocular angiogenesis. EMBO Mol. Med. 13:e12005. 10.15252/emmm.202012005, PMID: 33587337 PMC7933934

[ref33] Robinson M. D. McCarthy D. J. Smyth G. K. (2010). edgeR: a bioconductor package for differential expression analysis of digital gene expression data. Bioinformatics 26, 139–140. 10.1093/bioinformatics/btp616 19910308 PMC2796818

[ref34] Seguin F. Carvalho M. A. Bastos D. C. Agostini M. Zecchin K. G. Alvarez-Flores M. P.. (2012). The fatty acid synthase inhibitor orlistat reduces experimental metastases and angiogenesis in B16-F10 melanomas. Br. J. Cancer 107, 977–987. 10.1038/bjc.2012.355, PMID: 22892389 PMC3464771

[ref35] Smolock E. M. Korshunov V. A. Glazko G. Qiu X. Gerloff J. Berk B. C. (2012). Ribosomal protein L17, RpL17, is an inhibitor of vascular smooth muscle growth and carotid intima formation. Circulation 126, 2418–2427. 10.1161/CIRCULATIONAHA.112.125971, PMID: 23065385 PMC3502943

[ref36] Suzuki Y. Ito Y. Mizuno M. Kinashi H. Sawai A. Noda Y.. (2012). Transforming growth factor-beta induces vascular endothelial growth factor-C expression leading to lymph angiogenesis in rat unilateral ureteral obstruction. Kidney Int. 81, 865–879. 10.1038/ki.2011.464, PMID: 22258325

[ref37] Tamai K. Spaide R. F. Ellis E. A. Iwabuchi S. Ogura Y. Armstrong D. (2002). Lipid hydroperoxi destimulates subretinal choroidal neovascularization in the rabbit. Exp. Eye Res. 74, 301–308. 10.1006/exer.2001.1121, PMID: 11950240

[ref38] Tomita Y. Ozawa N. Miwa Y. Ishida A. Ohta M. Tsubota K.. (2019). Pemafibrate prevents retinal pathological neovascularization by increasing FGF21 level in a murine oxygen-induced retinopathy model. Int. J. Mol. Sci. 23:5878. 10.3390/ijms20235878, PMID: 31771164 PMC6928689

[ref39] Trapnell C. Williams B. A. Pertea G. Mortazavi A. Kwan G. Baren M. J.. (2010). Transcript assembly and quantification by RNA-Seq reveals unannotated transcripts and isoform switching during cell differentiation. Nat. Biotechnol. 28, 511–515. 10.1038/nbt.1621, PMID: 20436464 PMC3146043

[ref40] Walshe T. E. Saint-Geniez M. Maharaj A. S. Sekiyama E. Maldonado A. E. D'Amore P. A.. (2009). TGF-beta is required for vascular barrier function, endothelial survival and homeostasis of the adult microvasculature. PLoS One 4:e5149. 10.1371/journal.pone.0005149, PMID: 19340291 PMC2659748

[ref41] Wang X. Ma W. Han S. Meng Z. Y. Zhao L. Yin Y.. (2017). TGF-β participates choroid neovascularization through Smad2/3-VEGF/TNF-α signaling in mice with laser-induced wet age-related macular degeneration. Sci. Rep. 7:9672. 10.1038/s41598-017-10124-4 28852052 PMC5575286

[ref42] Weidner N. Carroll P. R. Flax J. Blumenfeld W. Folkman J. (1993). Tumor angiogenesis correlates with metastasis in invasive prostate carcinoma. Am. J. Pathol. 143, 401–409. PMID: 7688183 PMC1887042

[ref43] Xiao Y. L. Wu Y. Y. (2016). Interleukin-18 inhibits experimental choroidal neovascularization and its potential therapeutic applications. Chin. J. Ocul. Fundus Dis. 32, 457–459. 10.3760/cma.j.issn.1005-1015.2016.04.030

[ref44] Yang C. Chen W. Chen Y. Jiang J. (2012). Smoothened transduces hedgehog signal by forming a complex with Evc/Evc2. Cell Res. 22, 1593–1604. 10.1038/cr.2012.134, PMID: 22986504 PMC3494391

[ref45] Yang X. M. Wang Y. S. Xu J. F. X. Zhang P. (2006). Characteristics of choroidal neovascularization induced by laser in pigmented rats. Rec. Adv. Ophthalmol. 26, 161–166. 10.13389/j.cnki.rao.2006.03.001

[ref46] Zarranz-Ventura J. Fernández-Robredo P. Recalde S. Salinas-Alamán A. Borrás-Cuesta F. Dotor J.. (2013). Transforming growth factor-beta inhibition reduces progression of early choroidal neovascularization lesions in rats: P17 and P144 peptides. PLoS One 8:e65434. 10.1371/journal.pone.0065434, PMID: 23741494 PMC3669249

[ref47] Zhou T. Hu Y. Chen Y. Zhou K. K. Zhang B. Gao G. Q.. (2010). The pathogenic role of the canonical Wnt pathway in age ‐ related macular degeneration. Invest. Opthalmol. Vis. Sci. 51, 4371–4379. 10.1167/iovs.09-4278, PMID: 19875668 PMC2941176

[ref48] Zhu Z. R. Goodnight R. Sorgente N. Ogden T. E. Ryan S. J. (1989). Experimental subretinal neovascularization in the rabbit. Graefes Arch. Clin. Exp. Ophthalmol. 227, 257–262. 10.1007/BF02172759, PMID: 2472307

