# Supplementary figures and images for: Transcriptome Analysis of Choroid and Retina From Tree Shrew With Choroidal Neovascularization Reveals Key Signaling Moieties
Source: Front Genet. 2021 May 10;12:654955. doi: 10.3389/fgene.2021.654955 (PMC8141912; doi:10.3389/fgene.2021.654955)

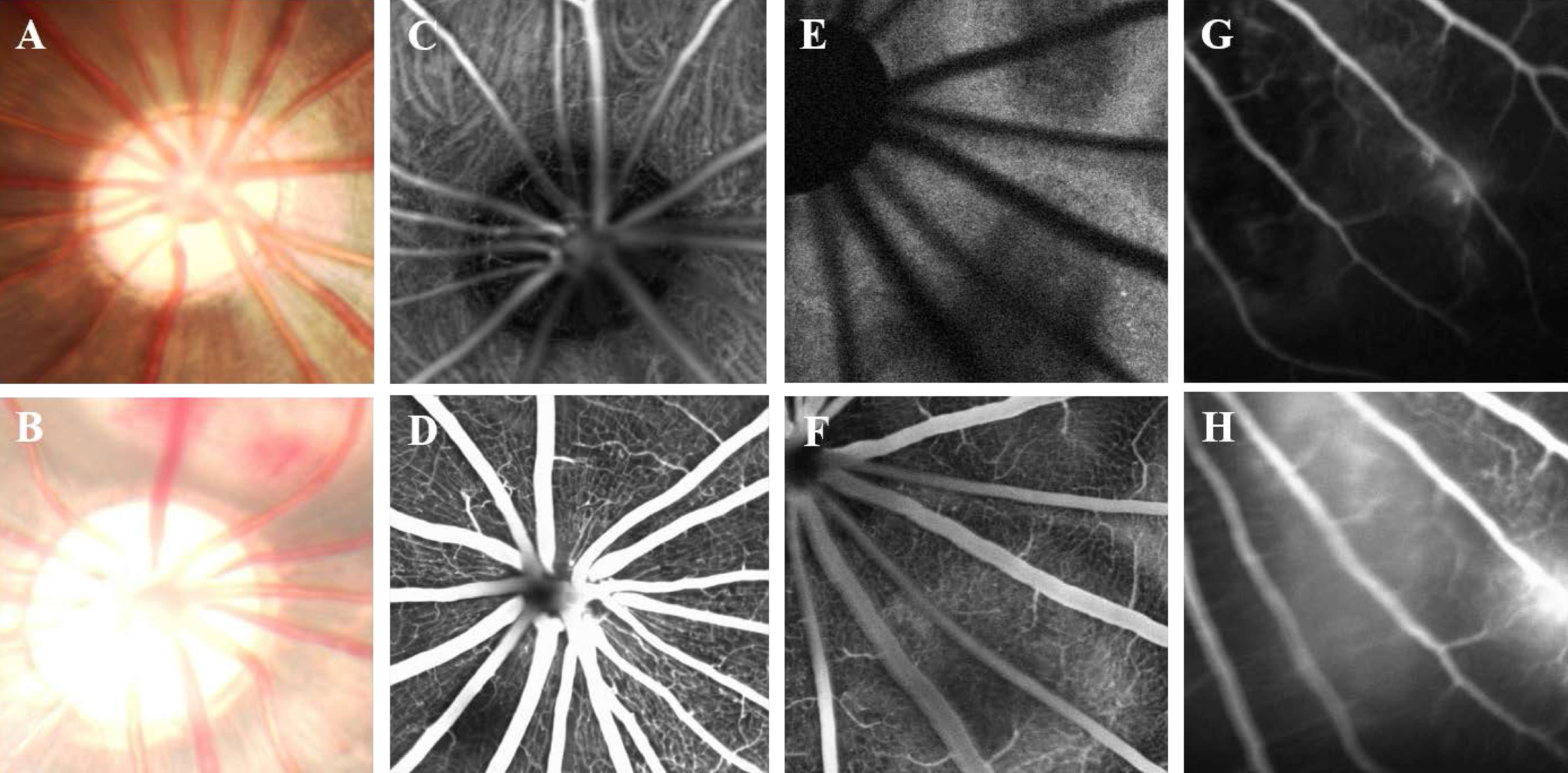

Supplement: Supplementary Figure 1 — Symptoms of tree shrews with choroidal neovascularization (CNV) after laser photocoagulation. (B) Representative fundus angiograph of normal tree shrew. (B) Representative fundus angiograph of tree shrew with laser photocoagulation. (C,D): FFA/ICGA of normal tree shrew. (E,F): FFA/ICGA of tree shrew after 7 days of laser photocoagulation. (G,H): FFA/ICGA of tree shrew after 30 days of laser photocoagulation. [file Image_1.tiff]
